# Cerebral, Muscle and Blood Oxygenation in Patients with Pulmonary Vascular Disease Whilst Breathing Normobaric Hypoxia vs. Normoxia Before and After Sildenafil: Data from a Randomised Controlled Trial

**DOI:** 10.3390/jcm14238407

**Published:** 2025-11-27

**Authors:** Alina Häfliger, Michael Furian, Simon R. Schneider, Julian Müller, Meret Bauer, Arcangelo F. Carta, Esther I. Schwarz, Stéphanie Saxer, Mona Lichtblau, Silvia Ulrich

**Affiliations:** 1 Pulmonology Department, University and University Hospital Zurich, 8091 Zurich, Switzerland; 2 MOVE-IT, Eastern Switzerland University of Applied Sciences, 9001 St. Gallen, Switzerland

**Keywords:** pulmonary hypertension, pulmonary vascular disease, physiology, cerebral autoregulation, altitude research

## Abstract

**Background**: In patients with pulmonary arterial hypertension or chronic thromboembolic pulmonary hypertension (summarized as pulmonary vascular disease; PVD), it is unclear whether the brain is protected against acute hypoxia and whether acute pulmonary vascular dilatation by sildenafil would influence cerebral and muscle tissue oxygenation whilst breathing normoxia or hypoxia. **Methods**: Adult patients with PVD underwent right heart catheterization, while cerebral and muscular tissue oxygenation and tissue hemoglobin index were measured using near-infrared spectroscopy along with arterial and mixed-venous blood gases. Participants underwent a four-stage protocol in which they were blinded to breathing either normoxia (FiO_2_ 0.21) or normobaric hypoxia (FiO_2_ 0.15), both before and after a single oral dose of sildenafil (50 mg) according to a randomized, cross-over design. **Results**: In 22 PVD patients (9 women, age 54 ± 14 y) under hypoxia, mean cerebral tissue oxygenation decreased by −2% (95% CI −4 to 0%, *p* = 0.046), muscular tissue oxygenation by −1% (95% CI −3 to 0%, *p* = 0.011) and mean arterial partial pressure of oxygen by −2.3 kPa (95% CI −2.7 to −1.8 kPa, *p* < 0.0001). Sildenafil improved the cerebral tissue hemoglobin index under hypoxia compared to hypoxia without sildenafil by 0.12 (95% CI 0.00 to 0.23, *p* = 0.049), but not the muscular tissue hemoglobin index. **Conclusions**: In PVD patients, acute exposure to normobaric hypoxia leads to a reduction in arterial oxygenation as well as cerebral and muscular tissue oxygenation. Sildenafil improves cerebral blood flow but has no effect on arterial, cerebral or muscular oxygenation.

## 1. Introduction

Adequate oxygen delivery to the brain is essential for cerebral function. Consequently, cerebral blood flow is modified in response to neuronal activity and humoral stimuli. Augmented neuronal activity leads to a local increase in cerebral blood flow. In contrast, humoral stimuli, especially a decrease in the arterial partial pressure of oxygen (PaO_2_) or an increase in the partial pressure of carbon dioxide (PaCO_2_), lead to a global rise in cerebral blood flow [1,2]. The use of near-infrared spectroscopy (NIRS) allows for the non-invasive measurement of cerebral tissue oxygenation saturation (CTO) [3,4], as well as the detection of impairments in cerebral autoregulation, which is a crucial aspect, as it can potentially lead to severe damage to cerebral tissue, such as cerebral ischemia [5,6]. Only a little is known about cerebral autoregulation in patients with pulmonary vascular disease (PVD) with precapillary pulmonary hypertension (PH) diagnosed as pulmonary arterial hypertension or chronic thromboembolic PH (PAH/CTEPH) [7,8]. The main symptom of PVD is exertional dyspnea, which is typically associated with oxygen desaturation [9]. Exposure to hypobaric hypoxia, such as at high altitude or during air travel, results in further deterioration of oxygen levels and, subsequently, cerebral hypoxia [10]. Current PH guidelines advise symptomatic patients not to travel to high altitudes without supplemental oxygen [7]. However, better treatment options have improved symptoms in some patients with PVD so that they wish to participate in social activities, including travel to high altitudes [11,12]. Therefore, an increased understanding of the effects of hypoxia exposure on physiology, including cerebral and muscular oxygenation and regional tissue perfusion, is important.

Sildenafil is a selective phosphodiesterase-5 inhibitor that causes vasodilation of the pulmonary arteries by enhancing the effect of nitric oxide [13,14]. It may therefore hamper hypoxic pulmonary vasoconstriction, which causes pulmonary artery pressure (PAP) to rise in healthy individuals and PVD patients, and to ameliorate pulmonary hemodynamic under hypoxia [15]. The effect of sildenafil on cerebral vasculature remains an understudied topic, particularly in the context of chronic pulmonary disease and hypoxia.

Therefore, the objective of the current randomized clinical trial was to investigate the patterns of cerebral and muscular tissue oxygenation saturation (CTO/MTO), as well as the tissue hemoglobin index (THI), as a substitute for regional blood flow, along with arterial and mixed venous blood oxygenation during right heart catheterization (RHC) in patients with PVD whilst breathing normoxia or hypoxia before and after a single oral dose of 50 mg sildenafil. We hypothesized that CTO and MTO would be reduced under hypoxia and might improve after sildenafil due to vasodilation-associated increased blood flow.

## 2. Materials and Methods

### 2.1. Design and Setting

This study was conducted as a part of a randomized controlled single-blind crossover trial assessing pulmonary hemodynamics in patients with PVD at rest and during exercise under normoxia (FiO_2_ 0.21) and normobaric hypoxia (FiO_2_ 0.15) before and after a single dose of sildenafil [16]. Between February 2021 and May 2023, patients with suspected PVD were scheduled for a diagnostic RHC at the University Hospital Zurich (470 m above sea level). Investigations during RHC included measurements at rest and during identical bouts of submaximal exercise while breathing normoxia and hypoxia, each before and after a 50 mg oral dose of sildenafil. The focus of this current study were the effects of hypoxia with and without sildenafil on CTO, MTO and cerebral and muscle THI (cTHI, mTHI) at rest, which has not yet been published.

The trial was approved by the local ethics committee (BASEC 2020-02163), registered at ClinicalTrials.gov (NCT04706546), and written informed consent was obtained from all participants.

### 2.2. Participants

Individuals referred for clinically indicated RHC who met the following criteria were eligible: signed informed consent, age 18 years or older, both sexes and PH groups I (PAH) or IV (CTEPH) diagnosed according to contemporary guidelines [17]. Excluded were patients with a resting PaO_2_ < 8 kPa on ambient air, who were exposed to an altitude > 1000 m for ≥3 nights during the last 2 weeks before the study, who were unable to follow the study protocol, who took nitrates or had other clinically significant concomitant end-stage diseases such as renal failure or cancer.

### 2.3. Intervention, Randomization and Blinding

Patients were breathing either normobaric hypoxia (FiO_2_ 0.15) corresponding to a simulated altitude of ~2500 m or normoxia provided by an altitude simulator device (AltiTrainer^®^, SMTEC, Sport & Medical Technologies SA, 8451 Kleinadelfingen, Switzerland) through a face mask in a randomized order during a supine rest. Measurements were taken after 15 min of application time. The wash-out period between the two interventions was at least 15 min. The identical sequences were repeated starting from 50 min after a 50 mg oral dose of sildenafil. Allocation to one of the four possible study arms was performed by a computer algorithm. Patients were fully blinded to the gas mixture but not to the vasodilator. Investigators were not blinded as SpO_2_ was monitored for safety reasons. Data analysis was performed by investigators blinded to the interventions.

### 2.4. Assessments

#### 2.4.1. Baseline Medical History

Demographics, current medication and New York Heart Association functional class (NYHA) were assessed. Spirometry and single-breath carbon monoxide diffusing capacity, 6-min walking distance and venous blood sampling were obtained at the same appointment of the RHC or from a recent outpatient visit.

#### 2.4.2. Cerebral and Muscle Tissue Oxygenation by NIRS

CTO and MTO were assessed using NIRS (NIRO 200NX, Hamamatsu, Japan) emitting light at 735, 810 and 850 nm. Optodes were placed on the forehead and the quadriceps muscle [6,18]. The relative concentration of oxygenated (O_2_Hb) and deoxygenated (HHb) hemoglobin was measured, and CTO and MTO, respectively, were calculated in percentage as

(1) $$\frac{\left(O_{2}Hb\right)}{\left(O_{2}Hb+HHb\right)}\times 100.$$

In addition, total Hb (totHb), the sum of O_2_Hb and HHb, was computed to determine the THI, a surrogate for regional blood flow changes [19,20,21].

#### 2.4.3. Blood Gas Analysis and Oximetry

A radial artery line was placed. Arterial (a) and mixed venous (v) blood gas analysis, including Hb, pO_2_, pCO_2_, SO_2_, pH and lactate, were measured from radial artery and pulmonary artery samples, respectively (Siemens RAPIDPoint^®^ 500 analysator, 91301 Forchheim, Germany).

The arterial oxygen content (CaO_2_) was calculated using the following formula [22]:

CaO_2_ = SaO_2_ × Hb × 1.37 + PaO_2_ × 0.0031. (2)

#### 2.4.4. Right Heart Catheterization and Metabolic Measurements

A Swan-Ganz catheter (Swan Ganz CCOmbo V, Edwards Lifesciences, Irvine, CA 92614, USA) was placed in the pulmonary artery via the right jugular vein under sonographic control. The pressure transducers were placed at 50% of the back-sternum distance at the presumptive level between the right and left atrium and zeroed to atmospheric pressure [23]. The following parameters were assessed: heart rate, systemic arterial pressure (systolic, diastolic, mean blood pressure (BP)), PAP (systolic, diastolic, mean PAP), pulmonary arterial wedge pressure (PAWP) and right atrial pressure (RAP). Metabolic measurements were performed at rest with breath-by-breath recordings of ventilation (minute ventilation, tidal volume, breath rate) and gas exchange (VO_2_, VCO_2_). The above-mentioned physiological parameters were averaged over 30 s at the end of each step of the protocol.

Cardiac output (CO) was calculated using the direct Fick method [24]:

(3) $$CO=\frac{VO_{2}}{{CaO}_{2}-{CvO}_{2}}.$$

To determine the pulmonary vascular resistance (PVR) and the systemic vascular resistance (SVR), the following formulas were utilized:

(4) $$\mathrm{PVR}\;=\;\frac{mPAP-PAWP}{CO},$$

(5) $$\mathrm{SVR}=\frac{BP-RAP}{CO}.$$

#### 2.4.5. Outcomes

The outcomes of this analysis were changes in CTO and cTHI under hypoxia, without and with sildenafil. Further outcomes included changes in MTO, mTHI and other hemodynamic parameters, such as SaO_2_, heart rate and blood pressure.

### 2.5. Statistical Analysis

Data analysis was performed in the per-protocol population, including all patients who completed all four phases according to the protocol. All data are summarized as the mean ± standard deviation or standard error and mean difference with 95% CI. Outcomes were analyzed using a linear mixed model, including a random intercept for the participant. The model was adjusted for each allocation sequence to check for the carry-over effect. Missing values were handled via listwise deletion. A *p*-value < 0.05 was considered statistically significant. All statistical analyses were performed with R Studio (version 4.3.1, RStudio Inc., San Francisco, CA, USA) using the lmerTest and emmeans packages.

## 3. Results

**Figure 1:**
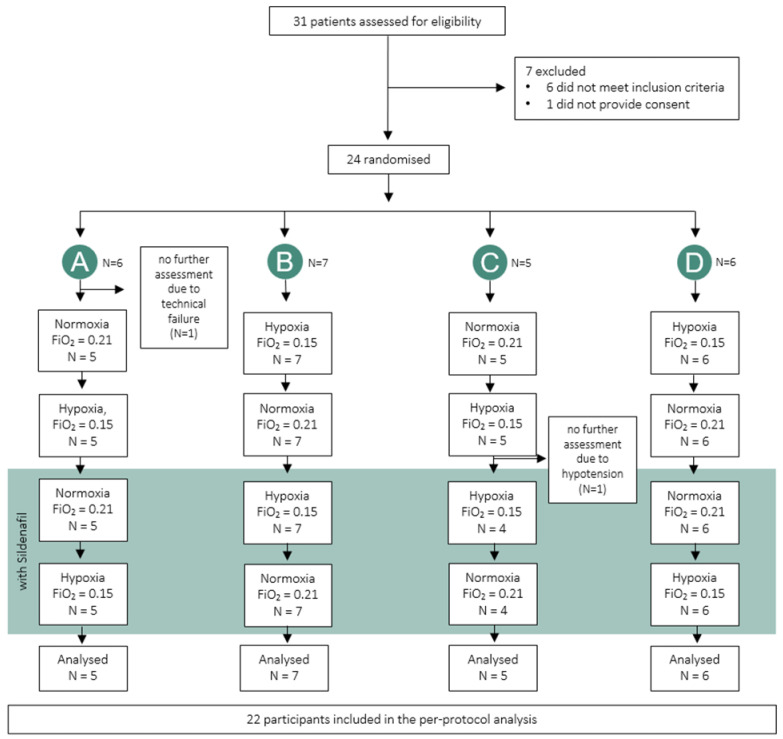
Patient flowchart.

Of the 31 PVD patients assessed for eligibility, 7 were excluded because they did not meet the inclusion criteria. Twenty-four patients were randomized to one of the four study arms, of which two patients had to be excluded after randomization due to technical failure or hypotension (Figure 1). Consequently, 22 patients with PVD were included in this per-protocol analysis. Baseline patient characteristics are presented in Table 1. Most patients with PAH or CTEPH were classified as NYHA II and were pre-treated with PH-targeted medication.

**Figure 2:**
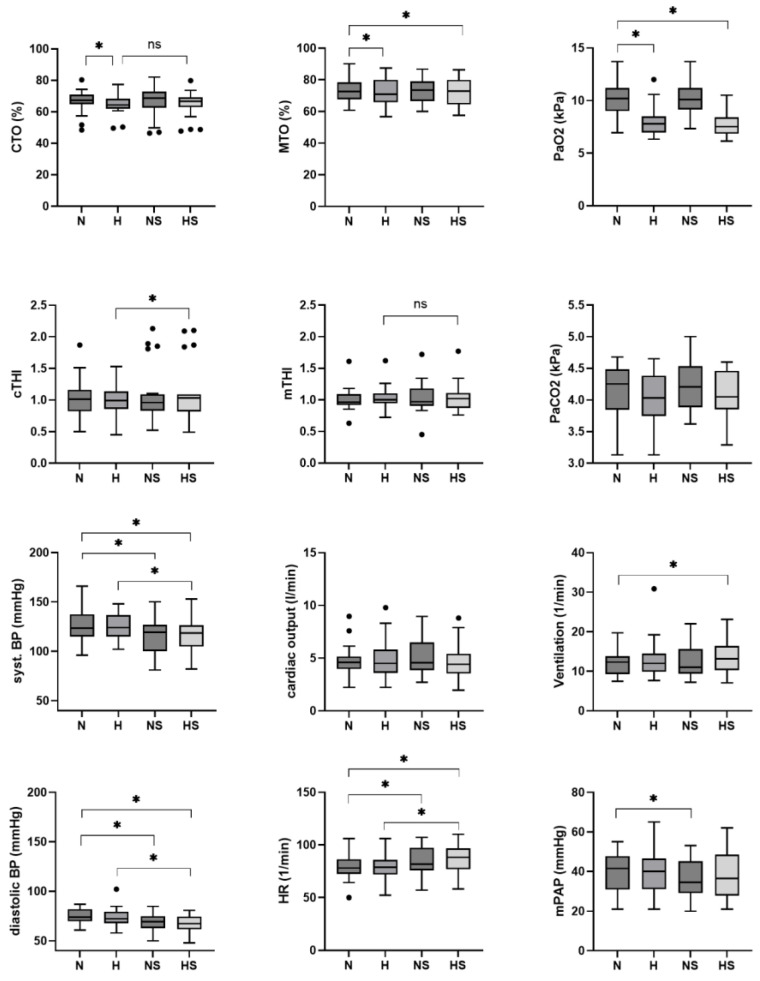
Outcome measured at rest and supine after 10 min of normoxia and hypoxia, before and after 50 mg of additive oral sildenafil. * shows significant changes, *p* < 0.05. ns: non-significant changes. N: normoxia. H: hypoxia. NS: normoxia and sildenafil. HS: hypoxia and sildenafil. CTO: cerebral tissue oxygenation. cTHI: cerebral tissue hemoglobin index. MTO: muscular tissue oxygenation. mTHI: muscular tissue hemoglobin index. HR: heart rate. BP: blood pressure. mPAP: mean pulmonary artery pressure. PaO_2_: arterial oxygen partial pressure. PaCO_2_: arterial carbon dioxide partial pressure.

The mean CTO decreased by 2% (95% CI −4 to 0%, *p* = 0.046) under hypoxia, and sildenafil had no detectable effect on the hypoxia-related reduction in CTO (mean difference of 0%, 95% CI −2 to 2%, *p* = 0.707). Sildenafil improved cTHI under hypoxia when compared to hypoxia without sildenafil by 0.12, 95% CI 0.00 to 0.23, *p* = 0.049. This effect was not seen for mTHI (mean difference of 0.01, 95% CI −0.04 to 0.07, *p* = 0.666), although MTO decreased under hypoxia by 1%, 95% CI −3 to 0%, *p* = 0.011 (Table 2, Figure 2).

Under hypoxia, PaO_2_ decreased by 2.3 kPa (*p* < 0.001) without sildenafil and by 2.6 kPa (*p* < 0.001) with sildenafil, both compared to normoxia. Only a slight decrease in PaCO_2_ was observed under hypoxia, with a mean change of −0.1 kPa, with no change after sildenafil. CaO_2_ was reduced under hypoxia vs. normoxia to a similar degree before and after sildenafil. The arterial pH increased under hypoxia compared with normoxia, but this was not significantly different after sildenafil.

The respiratory rate and CO remained unchanged under hypoxia vs. normoxia, before and after sildenafil (Table 2). Systemic hemodynamics were unchanged by hypoxia, but after sildenafil, blood pressure was reduced in normoxia and hypoxia to a similar degree. As a consequence, heart rate increased by 5 bpm after sildenafil in normoxia and hypoxia.

## 4. Discussion

This is the first study to investigate cerebral, muscular, arterial and mixed venous oxygenation in patients with PAH or CTEPH upon acute exposure to normobaric hypoxia vs. normoxia, before and after a single oral dose of 50 mg sildenafil. We found that hypoxia decreased arterial and mixed venous oxygen content to a similar degree before and after sildenafil. CTO was also reduced under hypoxic conditions, and although sildenafil improved cTHI, a surrogate marker for cerebral blood flow, it was unable to prevent the reduction in CTO under hypoxia. Likewise, MTO decreased under hypoxia, but sildenafil was not able to improve mTHI.

Despite lower blood and tissue oxygenation, our cohort of PVD patients did not appear to be stressed sufficiently by this short-term exposure, which was reflected by an unchanged heart rate or respiratory rate under hypoxia compared to normoxia. A possible explanation for this lack of response could be that the stimulus itself was too weak and that the decline in blood oxygenation was too mild to elicit expected physiological reactions at rest. The other explanation could be that this PVD cohort did not adequately react to this stimulus due to adaptation to an underlying respiratory disease. A comparison of the mean PaO_2_ of these PVD patients with that of a healthy population at an altitude of 2500 m reveals a lower mean PaO_2_ in PVD patients, which is still within the normal range of PaO_2_, as found at altitude [25]. Although Forrer et al. looked at values under hypobaric hypoxia, it can be assumed that PVD patients desaturated within the expected range. In a control group without PVD, it was observed that healthy participants desaturated more than PVD patients since they started at a higher PaO_2_ [22]. Moreover, an increase in heart rate was observed in both groups, with a more pronounced hyperventilatory response in PVD patients.

To the best of our knowledge, no study with a similar study setup exists that investigates CTO and cTHI as substitutes for regional blood flow under hypoxia in healthy individuals; thus, we have no comparative group.

As expected, PVD patients reacted to sildenafil with a decreased systemic blood pressure, a compensatory increase in heart rate, a decrease in mPAP and unchanged SpO_2_ [26]. Under hypoxia, SpO_2_ declined regardless of sildenafil. Poudel et al. observed that there are no significant differences in SpO_2_ between subjects receiving sildenafil or a placebo despite a reduction in PAP in normobaric hypoxia in a healthy population at rest [13]; our results extend these findings to patients with PVD. The authors propose that this can be explained by hypoxic pulmonary vasoconstriction (HPV), a protective mechanism to redistribute blood from poorly ventilated lung regions to better ventilated lung regions [27]. Patients with PVD seem to be protected from HPV due to their already altered pulmonary vasculature [28]. In our study, sildenafil did not further affect blood gases, suggesting that it did not negatively affect gas exchange in PVD [10].

Our results show that sildenafil was able to increase the cTHI. This suggests that sildenafil may cause vasodilatation of cerebral vessels. Moreover, this effect persisted during hypoxia. Yet, this vasodilatation during hypoxia was unable to prevent the decline in CTO. A previous study in infants after cardiac surgery described an increase in cerebral blood flow after intravenous administration of sildenafil using NIRS [29]. Another study performed in healthy adults at high altitude showed enhanced cerebral oxygenation following oral administration of 50 mg sildenafil [30]. Additionally, they describe an interesting mechanism that may explain our findings of increasing THI and decreasing CTO. It has been proposed that the proportion of arterial and venous blood in the capillary bed may change under hypoxic conditions and in response to sildenafil [31,32]. However, both studies had a completely different study setup and study population, which makes it difficult to compare their results to our adult PVD population. Nevertheless, it gives us some insights into the potential mechanism of sildenafil on cerebral blood flow as measured by NIRS.

Other drugs have been shown to improve CTO in PVD- patients. For example, acetazolamide improved CTO in PVD patients under normobaric hypoxia [33]. The authors stated that this was most likely due to the known direct vasodilator effect of acetazolamide induced by the lowered arterial pH and a direct inhibition of carbonic anhydrase in brain vessels. Another study using nitric oxide as a vasodilator showed that compared to healthy controls, CTO was improved in PVD patients after administring nitric oxide [34]. This study did not test nitric oxide in PVD patients under hypoxia, only in normoxia. Neither study showed an improvement in THI, but only in CTO. This is probably due to the fact that both interventions resulted in improved global oxygenation.

### Limitations

As this was a crossover trial, there is a risk of carry-over effects. This was ruled out by an outcome adjustment for our allocation sequence, which did not reveal a difference. Exposure to hypoxia in our study was short due to the complexity of the study setup, with four sequences, which in itself required time for participants in the RHC laboratory. More distinct results could have been achieved if we had extended the exposure to hypoxia or used a lower FiO_2_. This study was performed in normobaric hypoxia, but there might be a small difference compared to hypobaric hypoxia [35]. This makes it challenging to make statements regarding travel fitness for high altitudes. However, our results provide insights into the pathophysiological mechanisms. Further trials are warranted to study the additive effects of sildenafil in real-life high-altitude conditions. Finally, we used optodes measuring CTO and cTHI in the prefrontal cortex, which is important for cognitive functions [36]. Whether these results apply to other regions of the brain remains open.

## 5. Conclusions

In conclusion, this study in patients with PAH or distal CTEPH revealed that, along with the expected lower arterial and mixed venous oxygen content under acute exposure to normobaric hypoxia vs. normoxia, CTO and MTO were also lowered. Furthermore, we observed that sildenafil improved cTHI, a surrogate marker of cerebral blood volume, but did not prevent the decline in CTO under hypoxia. The results of this study contribute to the increasing evidence that sildenafil has a direct effect on the cerebral vasculature. However, the precise mechanism by which this occurs remains to be elucidated through further investigation.

## Figures and Tables

**Table 1:** Patient characteristics.

Variable N = 22	Value
Sex, ♀/♂ (%)	9/13 (41/59)
Age, y	54 ± 14
Body mass index, kg/m^2^	26.3 ± 4.0
New York Heart Association functional class I, II, III, IV (%)	1 (4), 16 (73), 5 (23), 0 (0)
6-min walk distance, m	553 ± 78
**Pulmonary hypertension classification, n (%)**	
Pulmonary arterial hypertension	14 (64)
Idiopathic/hereditary	12
Connective tissue disease	1
Portal hypertension	1
Chronic thromboembolic pulmonary hypertension	8 (36)
**Pulmonary function and hemodynamic**	
Resting PaO_2_, kPa	9.6 ± 1.2
Mean pulmonary arterial pressure, mmHg	40 ± 11
Pulmonary vascular resistance, Wood units	6.0 ± 2.7
**Pulmonary hypertension targeted therapy, n (%)**	
Endothelin receptor antagonist	9 (41)
Phosphodiesterase-5 inhibitor	9 (41)
Soluble guanylate cyclase stimulators	3 (14)
Prostacyclin receptor agonist or prostacyclin	6 (27)
Combination therapy	8 (36)
No PH-specific therapy	8 (36)

Data are shown as the mean ± standard deviation or number (proportions). PaO_2_: arterial oxygen partial pressure; PH: pulmonary hypertension.

**Table 2:** Outcome measures at rest supine during normoxia and hypoxia before and after 50 mg of additive oral sildenafil (corrected for randomization).

	Normoxia (N)	Hypoxia (H)	Normoxia After Sildenafil (NS)	Hypoxia After Sildenafil (HS)	Mean Difference ∆ H-N (95% CI)	Sildenafil Effect on Normoxia, Mean Difference ∆ NS-N (95% CI)	Mean Difference ∆ HS-NS (95% CI)	Sildenafil Effect on Hypoxia, Mean Difference ∆HS-H (95% CI)
**Tissue oxygenation**								
CTO, %	66 ± 2	64 ± 2	67 ± 2	65 ± 2	−2 (−4 to 0) *	0 (−2 to 2)	−2 (−4 to 0) *	0 (−2 to 2)
cTHI	1.02 ± 0.09	0.99 ± 0.09	1.09 ± 0.09	1.11 ± 0.09	−0.02 (−0.14 to 0.09)	0.07 (−0.04 to 0.19)	0.03 (−0.09 to 0.14)	0.12 (0.00 to 0.23) *
MTO, %	73 ± 2	72 ± 2	72 ± 2	72 ± 2	−1 (−3 to 0) *	−1 (−2 to 0)	−1 (−2 to 0)	0 (−1 to 1)
mTHI	1.01 ± 0.05	1.04 ± 0.05	1.04 ± 0.05	1.05 ± 0.05	0.03 (−0.02 to 0.09)	0.03 (−0.02 to 0.09)	0.01 (−0.04 to 0.07)	0.01 (−0.04 to 0.07)
**Blood gas analysis and Ventilation**								
Hemoglobin, g/dL	15.4 ± 0.3	15.5 ± 0.3	15.4 ± 0.3	15.4 ± 0.3	0.1 (−0.1 to 0.2)	0.0 (−0.2 to 0.1)	0.0 (−0.1 to 0.2)	−0.1 (−0.2 to 0.1)
Lactate, mmol/L	0.9 ± 0.1	0.7 ± 0.1	1.4 ± 0.1	1.2 ± 0.1	−0.1 (−0.3 to 0.0)	0.5 (0.3 to 0.7) *	−0.2 (−0.4 to 0.0)	0.5 (0.3 to 0.6) *
pHa	7.45 ± 0.01	7.47 ± 0.01	7.44 ± 0.01	7.46 ± 0.01	0.01 (0.00 to 0.03) *	−0.01 (−0.02 to 0.00)	0.01 (0.00 to 0.02) *	−0.01 (−0.02 to 0.00)
PaO_2_, kPa	10.1 ± 0.3	7.9 ± 0.3	10.1 ± 0.3	7.6 ± 0.3	−2.3 (−2.7 to −1.8) *	0.0 (−0.5 to 0.5)	−2.6 (−3.0 to −2.1) *	−0.3 (−0.8 to 0.2)
PaCO_2_, kPa	4.1 ± 0.1	4.0 ± 0.1	4.2 ± 0.1	4.1 ± 0.1	−0.1 (−0.3 to 0.0)	0.1 (−0.1 to 0.3)	−0.2 (−0.3 to 0.0) *	0.0 (−0.1 to 0.2)
SaO_2_, %	94 ± 1	90 ± 1	95 ± 1	90 ± 1	−4 (−5 to −3) *	0 (−1 to 2)	−5 (−6 to −4) *	−1 (−2 to 0)
CaO_2_, mL/L	193 ± 4	185 ± 4	192 ± 4	182 ± 4	−8 (−11 to −5) *	−1 (−4 to 2)	−10 (−13 to −7) *	−3 (−6 to 0)
PmvO_2_, kPa	4.9 ± 0.1	4.5 ± 0.1	4.9 ± 0.1	4.6 ± 0.1	−0.3 (−0.5 to −0.2) *	0.1 (−0.1 to 0.2)	−0.3 (−0.5 to −0.2) *	0.0 (−0.1 to 0.2)
PmvCO_2_, kPa	4.8 ± 0.1	4.7 ± 0.1	4.9 ± 0.1	4.6 ± 0.1	−0.1 (−0.3 to 0.1)	0.1 (0.0 to 0.3)	−0.3 (−0.4 to −0.1) *	0.0 (−0.2 to 0.1)
SvO_2_, %	67 ± 1	63 ± 1	65 ± 1	63 ± 1	−4 (−7 to −1) *	−2 (−4 to 1)	−2 (−5 to 0)	0 (−3 to 3)
**Hemodynamics**								
Heart rate, bpm	78 ± 3	79 ± 3	84 ± 3	87 ± 3	1 (−2 to 4)	5 (2 to 9) *	3 (0 to 6)	8 (5 to 11) *
Syst. BP, mmHg	128 ± 4	126 ± 4	115 ± 4	117 ± 4	−2 (−7 to 2)	−13 (−18 to −9) *	2 (−3 to 6)	−9 (−13 to −4) *
Dia. BP, mmHg	75 ± 2	75 ± 2	69 ± 2	67 ± 2	−1 (−4 to 2)	−7 (−9 to −4) *	−1 (−4 to 1)	−7 (−10 to −5) *
Respiratory Rate, 1/min	12 ± 1	13 ± 1	12 ± 1	14 ± 1	1 (0 to 2)	0 (−1 to 2)	1 (0 to 2)	1 (−1 to 2)
Mean PAP, mmHg	39 ± 2	40 ± 2	36 ± 2	38 ± 2	1 (−2 to 3)	−3 (−6 to −1) *	2 (−1 to 4)	−2 (−5 to 0)
Cardiac output, L/min	4.7 ± 0.4	4.9 ± 0.4	5.1 ± 0.4	4.7 ± 0.4	0.2 (−0.5 to 0.8)	0.4 (−0.3 to 1.0)	−0.4 (−1.1 to 0.2	−0.2 (−0.9 to 0.5)
SVR, WU	20.6 ± 1.7	20.7 ± 1.7	18.0 ± 1.7	19.0 ± 1.7	0.1 (−2.0 to 2.3)	−2.6 (−4.8 to −0.5) *	1.0 (−1.1 to 3.2)	−1.7 (−3.9 to 0.5
PVR, WU	7.10 ± 0.81	7.16 ± 0.82	5.99 ± 0.82	6.75 ± 0.81	0.06 (−0.92 to 1.05)	−1.11 (−2.09 to −0.13) *	0.76 (−0.22 to 1.74)	−0.42 (−1.40 to 0.57)

Data are shown as the mean ± SE or mean (CI 95%). * indicates statistical significance. CTO: cerebral tissue oxygenation. cTHI: cerebral tissue hemoglobin index. MTO: muscular tissue oxygenation. mTHI: muscular tissue hemoglobin index. BP: blood pressure. PAP: pulmonary artery pressure. CO: cardiac output measured with the Fick method. SVR: systemic vascular resistance ((MBP-RAP)/CO). PVR: pulmonary vascular resistance ((mean PAP-PAWP)/CO). PaO_2_: arterial oxygen partial pressure. PaCO_2_: arterial carbon dioxide partial pressure. SaO_2_: arterial oxygen saturation. CaO_2_: arterial oxygen concentration. PmvO_2_: mixed venous oxygen partial pressure. PmvCO_2_: mixed venous carbon dioxide partial pressure. SvO_2_: venous oxygen saturation.

## Data Availability

The data presented in this study are available on request from the corresponding author.

## Abbreviations

The following abbreviations are used in this manuscript:

BP	blood pressure
CaO_2_	arterial oxygen content
CTEPH	chronic thromboembolic pulmonary hypertension
cTHI	cerebral tissue hemoglobin index
CTO	cerebral tissue oxygenation saturation
HHb	relative concentration of deoxygenated hemoglobin
HPV	hypoxic pulmonary vasoconstriction
mTHI	muscular tissue hemoglobin index
MTO	muscular tissue oxygenation saturation
NIRS	near-infrared spectroscopy
NYHA	New York Heart Association
O_2_Hb	relative concentration of oxygenated hemoglobin
PAH	pulmonary arterial hypertension
PaCO_2_	arterial partial pressure of carbon dioxide
PaO_2_	arterial partial pressure of oxygen
PAP	pulmonary artery pressure
PAWP	Pulmonary arterial wedge pressure
PH	pulmonary hypertension
PVD	pulmonary vascular disease
RAP	right atrial pressure
RHC	right heart catheterization
SpO_2_	peripheral oxygen saturation
SVR	systemic vascular resistance
totHb	sum of O_2_Hb and HHb
